# Is areca innocent? The effect of areca (betel) nut chewing in a population of pregnant women on the Thai–Myanmar border

**DOI:** 10.1016/j.inhe.2012.05.001

**Published:** 2012-09

**Authors:** Amy L. Chue, Verena I. Carrara, Moo Kho Paw, Mupawjay Pimanpanarak, Jacher Wiladphaingern, Michele van Vugt, Sue J. Lee, François Nosten, Rose McGready

**Affiliations:** a Shoklo Malaria Research Unit, 68/30 Baan Tung Road, P.O. Box 46, Mae Sot, Tak 63110, Thailand; b Faculty of Tropical Medicine, Mahidol University, 420/6 Ratchawithi Road, Ratchathewi, Bangkok 10400, Thailand; c Centre for Clinical Vaccinology and Tropical Medicine, Churchill Hospital, Oxford OX3 7LJ, UK

**Keywords:** Areca nut, Smoking, Pregnancy, Maternal outcome, Neonatal outcome

## Abstract

Eight manuscripts have specifically examined the effects of areca (betel) nut use in pregnant women, seven of which have documented adverse effects on birth weight, newborn neurological status, gender ratio and pregnancy outcomes such as anaemia and miscarriage following areca nut use during pregnancy. A retrospective cohort analysis of migrant and refugee pregnant women attending antenatal clinics along the Thai–Myanmar border (July 1997 to November 2006) was conducted to examine the adverse effects of areca nut use routinely recorded on enrolment. Of 7685 women, 2284 (29.7%) never used areca or smoked (cheroots), 2484 (32.3%) only used areca, 438 (5.7%) only smoked cheroots and 2479 (32.3%) used both areca and cheroots. Pieces of ripe areca nut in a leaf with lime, without tobacco, were used particularly among older multigravid women. Adverse pregnancy effects were not observed in areca nut users compared with non-users. Smoking, but not areca nut use, had a dose-related effect on miscarriage. Areca nut use in conjunction with smoking reduced the adverse effects of smoking on birth weight, further supporting a lack of effect of areca nut. Areca (betel) nut-related adverse pregnancy outcomes were not observed in this population, whereas smoking was clearly harmful. Differences from previous reports may result from the amount or types of areca nut, or quid content, consumed between countries. Smoking, but not areca nut, reduction is likely to improve pregnancy outcomes on the Thai–Myanmar border.

## 1 Introduction

Areca (*Areca catechu* Linn.) nut is the fourth most common psychoactive substance in the world, with its use extending to 10–20% of the world's population.^1^ The nut itself contains alkaloids, including arecoline,^2^ which is thought to be the main contributor to the nut's stimulatory and toxic effects as described in human studies.^3^, ^4^ Slaked lime acts as a catalyst, enhancing the stimulatory effects of the nut.^5^ Areca nut with slaked lime (calcium hydroxide) wrapped in a *Piper betel* Linn. leaf (betel vine; family Piperaceae) forms a quid. The contents of the quid are important in terms of health effects as the contents vary remarkably between individuals locally^6^ and between countries^7^ and also depend upon the maturity of the areca nut, with the ripe nut having a higher concentration of arecoline (0.12–0.24%) compared with the unripe nut (0.11–0.14%).^4^, ^8^, ^9^, ^10^, ^11^, ^12^, ^13^ Spitting or swallowing of the quid will also result in different effects on health. ‘Betel nut’ is botanically incorrect and confusing. This manuscript will refer to areca nut, which is the seed of the fruit of the oriental palm *A. catechu* Linn*.*^8^

Carcinoma and dental problems are a known consequence of areca nut chewing,^14^, ^15^ with animal data suggesting that areca nut is teratogenic.^16^ In a review of the literature, eight manuscripts specifically examined the effects of areca nut use in a total of 1641 pregnant women.^4^, ^9^, ^10^, ^11^, ^12^, ^13^, ^17^, ^18^ Most manuscripts (5/7; 71%) reported low birthweight (LBW)^9^, ^10^, ^12^, ^13^, ^17^ and 2 (25%) reported reduced birth length.^13^, ^17^ Three measured haemoglobin: one reported a reduction in the frequency of maternal anaemia with areca nut use^11^ and two found no significant effect.^9^, ^10^ A higher maternal body mass index, reduced weight gain during pregnancy and a reduced male newborn rate were also found, but only in one paper.^13^ There were variable reports on neonatal withdrawal.^4^, ^9^ Arecoline was detected in the placenta of mothers chewing areca nut in six selected cases.^4^ No paper described a dose–response effect on birth weight. Consumption of areca nut remains common in the rural communities along the Thai–Myanmar border.^15^ The aim of this study was to provide more information on the effects of areca nut use in pregnancy and to investigate the consequences of areca nut chewing in refugee and migrant pregnant women and their newborns.

## 2 Methods

Mae La refugee camp, 60 km north of Mae Sot in Thailand, is the largest refugee camp for displaced people, mostly of Karen ethnicity from Myanmar, along the Thai–Myanmar border. Since 1986, Shoklo Malaria Research Unit (SMRU) (Mae Sot, Thailand) has encouraged women to attend antenatal clinics (ANC) for early detection and treatment of malaria owing to a lack of effective preventative methods and where prevention is complicated by multidrug-resistant *Plasmodium falciparum.*^19^ In 1998, ANC care commenced for migrant women who live along the border (but outside the camps). ANCs are open to all women as soon as they are aware of their pregnancy. The epidemiology of malaria and anaemia and their relation to birth outcomes in this population are described in detail elsewhere.^19^, ^20^ The clinics are staffed by local health workers. This study used the records of the ANCs of SMRU.

Smoking (not commercial cigarettes but cheroots) and areca nut use in pregnancy were recorded at the first antenatal visit. Women who smoked daily or more than once daily were classified as heavy smokers and those who did not smoke every day as light users. Women who consumed areca nut daily or more than once daily were classified as heavy users and those who did not take areca nut every day were light users. Women smoke cheroots and most commonly ‘Maw Htoo Wah’ (local Karen) or ‘Kye Lah’ (in Burmese), a 15 cm length of white dried betel leaf wrapper filled with coarse ground tobacco and a filter made from ‘Ahm way’, a blended herb that gives a sweet taste to the smoke. The cheroots have previously been analysed by the Laboratory of the Government Chemist (UK), revealing a high carbon monoxide yield (114 mg per cheroot) and a very high nicotine yield (3.8 mg per cheroot).^21^ Women were not routinely asked at enrolment about the use of alcohol or illicit drugs, as sale of alcohol is officially forbidden in Mae La refugee camp, alcohol abuse is rare and illicit drug use is even more uncommon as punishment in the past was severe.

The first antenatal consultation includes an obstetric and medical history and a detailed clinical examination for all women. Recording of maternal height was not routine until 2006. A body mass index of <18 kg/m^2^ only in women who attended in the first trimester was used to identify moderate or severe malnutrition. At each consultation, women were provided with 1 week of thiamine (vitamin B1, 100 mg daily), as infantile beriberi was previously the main cause of infant mortality.^22^ Haematocrit (Hct) samples were taken to screen for anaemia (Hct < 30%) and, if present, anaemia treatment was commenced, otherwise prophylaxis with ferrous sulphate and folic acid were provided. Hypertensive disorders were grouped together and included pregnancy-induced hypertension, eclampsia and pre-eclampsia, and chronic hypertension. Women were encouraged to follow-up weekly and to deliver with trained midwives in the SMRU delivery unit instead of giving birth at home with a traditional birth attendant. Caesarean section when needed was at the nearest Thai public hospital. All newborns were weighed using a Salter scale accurate to 50 g (Salter, Birmingham, UK) and were examined by trained midwives, including those born at home. Pregnancy records have been routinely entered into a data recording system since 1987.

Ultrasound gestational age examination was established at SMRU in August 2001.^23^ Prior to this, gestational age was usually estimated by the Dubowitz gestational age examination,^24^ fundal height formula^25^ or last menstrual period if no other estimate was available. During July 1997 to July 2001, when the Dubowitz examination was the main predictor of gestation, it was routine for infants to have the newborn neurological examination.^26^

This was an historical cohort where all records of women attending the ANC over a 9-year period (July 1997 to November 2006), the period where areca nut use was detailed, were reviewed. Demographic and delivery data were extracted, including first and last weight, last Hct and malaria infection. Ethical approval for review of the antenatal records was provided by the Oxford Tropical Research Ethics Committee (reference: OXTREC 28-09).

A separate cohort of 513 pregnant women was interviewed in December 2010 as part of an audit of midwifery advice provided to pregnant women. To provide counselling on alcohol, smoking and areca nut use in pregnant women, midwives conducted a cross-sectional survey to detail how areca was consumed in known users.

### 2.1 Pregnancy outcomes

Pregnancy outcomes were recorded on the ANC card and were reported as live births (with or without congenital malformations), stillbirth, miscarriage or lost to follow-up. Lost to follow-up was defined as discontinuation of antenatal care before the outcome of pregnancy was known, usually as a result of leaving the study area. Birth weight and gender were also recorded. Birth weight analysis was confined to live born, congenitally normal, singleton infants weighed in the first 72 h of life. LBW was defined as a weight below 2500 g, miscarriage as a pregnancy ending before 28 weeks gestation, and stillbirth as a delivery from 28 weeks gestational age or ≥800 g birth weight in which the infant displays no sign of life (gasping, muscular activity, cardiac activity); this was chosen as no ventilatory support is available in the clinics. Preterm births were those delivered before 37 weeks gestation.

### 2.2 Statistical analysis

Data were analysed in SPSS for Windows V.14.0 (SPSS, Benelux Inc., Gorinchem, The Netherlands). Continuous normally distributed data were described as the mean ± SD and non-normally distributed data as the median and range. To understand the effects of areca nut, women were grouped into ‘neither’ (those who never used areca or smoked), ‘areca only’, ‘smoke only’ or ‘areca and smoke’ (those who used areca and smoked cheroots). Pairwise comparisons against women who never used areca or smoked were made using Student's *t*-test for normally distributed data and the Mann–Whitney *U*-test for non-normally distributed data, and χ^2^ or Fisher's exact test for comparison of proportions, only if the fourway comparison was significant after Bonferroni adjustment.

Associations of areca nut use with anaemia (variables included in the model were smoking, users of both, multigravida, trimester of first ANC visit, anaemia at first ANC consultation and malaria); and with LBW (variables included in the model were primigravida, trimester of first consultation, malaria, gender of the newborn, smoking, gestational age at birth, mother's weight at delivery) were examined. Covariates that were significant at p < 0.10 were included in a multivariate logistic regression model where, using a stepwise approach, only those variables that were significant at p < 0.05 were retained in the final model and considered independent risk factors. Adjusted odds ratios were given with their 95% CI.

## 3 Results

### 3.1 Study population

A total of 9264 pregnant women were enrolled for antenatal care. There were 1289 women (13.9%) who moved from the study area before the outcome of their pregnancy was known and 290 (3.1%) had incomplete records for areca nut use or smoking, leaving 7685 (83.0%) evaluable pregnancies. There were 4963 women (64.6%) who reported areca nut use in pregnancy. Areca nut users had different characteristics than non-users (Table 1). Areca nut use, smoking and use of both were more common in multigravid women and in women of increasing age (p < 0.001 for both, linear trend; data not shown). Areca nut users were more likely to attend for their first consultation in the first trimester compared with non-users (Table 1).

**Table 1:** Characteristics of 7685 pregnant women attending antenatal care according to reported areca nut use and smoking in pregnancy

Population	Neither (n = 2284)	Areca only (n = 2484)	Smokers (n = 438)	Areca and smoke (n = 2479)	p-value^a^
Age (years) [mean ± SD (range)]^b^	24 ± 6 (14–46)	26 ± 6 (14–48)	27 ± 7 (15–46)	29 ± 7 (15–48)	1–3 *
Teenager (age <20 years) [n (%)]	679 (29.7)	435 (17.5)	53 (12.1)	172 (6.9)	1–3 *
Gravidity [median (range)]	2 (1–13)	3 (1–14)	3 (1–12)	4 (1–16)	1–3 *
Parity [median (range)]^c^	1 (0–10)	2 (0–11)	2 (0–10)	3 (0–13)	1–3 *
Primigravida [n (%)]	832 (36.4)	536 (21.6)	81 (18.5)	246 (9.9)	1–3 *
First ANC visit gestational age [median (range)]^d^	12 (–1 to 41)	11 (–2 to 42)	12 (0 to 40)	11 (–1 to 41)	1 *, 2^NS^, 3 **
First ANC visit in first trimester [n (%)]^e^	1383/2277 (60.7)	1611/2483 (64.9)	262/438 (59.8)	1603/2474 (64.8)	1 **, 2^NS^, 3 **
First maternal weight in first trimester (kg) [mean ± SD (range)]	47 ± 7 (31–98)	48 ± 7 (31–88)	46 ± 6 (21–66)	46 ± 6 (30–82)	1–2 **, 3 *
Mean maternal weight gain (kg) [mean ± SD (IQR)]	7.5 ± 4.2 (5–10)	7.2 ± 4.1 (5–10)	7.2 ± 3.7 (4–10)	7.0 ± 3.7 (5–9)	1–2^NS^, 3 **
BMI < 18 kg/m^2^ [n (%)]^f^	35/275 (12.7)	29/382 (7.6)	5/28 (17.9)	23/227 (10.1)	NS
Anaemic at first antenatal visit (first trimester women only) [n (%)]	83/1374 (6.0)	99/1601 (6.2)	20/262 (7.6)	134/1590 (8.4)	1–2^NS^, 3 **
Malaria during pregnancy [n (%)]	411 (18.0)	341 (13.7)	120 (27.4)	496 (20.0)	1–3 *
No. of consultations [median (range)]	24 (1–41)	22 (1–41)	19 (1–38)	22 (1–40)	1 *, 2 **, 3^NS^

ANC: antenatal clinic; NS: not significant; BMI: body mass index.

a p-value: ^1^neither vs areca only; ^2^neither vs smokers; ^3^neither vs areca and smoke.

b Age data not available for two women.

c Parity refers to the number of deliveries of live born and stillborn; parity data not available for four women.

d First ANC gestational age not available for 17 women and <1.0 for gestational age due to inherent error in gestational measurements.

e First ANC visit in first trimester not available for 13 women.

f BMI limited to a small number of women enrolled since 2006 and in first trimester (n = 912). * p < 0.001; ** p < 0.05.

Data on what women smoked was available for 86.2% of smokers (2514/2917) and, as previously reported,^21^ the majority (1377/2514; 54.8%) smoked Maw Htoo Wah.

### 3.2 Areca nut use

The proportion of women who used areca occasionally, daily and more than once daily was 84.2% (n = 2092), 10.0% (n = 249) and 5.8% (n = 143), respectively, in areca users alone, and 68.5% (n = 1697), 22.1% (n = 549) and 9.4% (n = 233), respectively, in users of both (p < 0.001, linear trend). The proportion of heavy (daily or more than once daily) areca nut use was significantly higher amongst women who also smoked (31.5%; 782/2479) compared with non-smokers (15.8%; 392/2484) (p < 0.001). There were 2284 women (29.7%) who never used areca or smoked, 2484 (32.3%) who only used areca, 438 (5.7%) who only smoked and 2479 (32.3%) who used areca and smoked cheroots.

### 3.3 December 2010 audit

Midwives surveyed 513 pregnant women in December 2010 and self-reported alcohol use in the current pregnancy included 10.3% (n = 53) of antenatal care attendees. Of these 53 women, 92.5% (n = 49) drank less than once a week. Areca nut use was reported by 55.0% (282/513) of women. Midwives reported that 86.2% of users (243/282) consumed areca nut after meals, 66.0% (186/282) without tobacco and 87.6% (247/282) in a *P. betel* Linn*.* leaf with slaked lime. Most women used less than one whole nut per day [53.9% (n = 152) compared with 31.6% (n = 89), 7.1% (n = 20) and 7.4% (n = 21) who used one to four, five to nine and ten or more whole nuts per day]. Tobacco use was more likely in women who used more than one whole areca nut per day compared with women who used less than one nut per day [56.9% (74/130) vs 14.5% (22/152); p < 0.001]. The majority of users (80.9%; 228/282) added slaked lime, spat initially and then swallowed the whole quid once disintegrated.

### 3.4 Pregnancy outcomes

Two women died before the outcome of pregnancy was known (severe malaria, ruptured uterus). There were 749 women (9.7%) who miscarried (Table 2). The higher proportion of miscarriage amongst smokers and users of both was dose related, with a higher frequency seen in heavy (16.4%; 146/890) compared with occasional smokers (11.9%; 241/2027; p = 0.001). In contrast, no dose–response relationship was observed for miscarriage for heavy (9.7%; 114/1174) compared with occasional areca nut users (10.6%; 400/3789; p = 0.443).

**Table 2:** Birth outcomes for women who use neither (areca or smoke cheroots) compared with areca only users, smokers and users of both

Population	Neither	Areca only	Smokers	Areca and smoke	p*-*value^a^
Miscarriage [n (%)]	176/2283^b^ (7.7)	187/2484 (7.5)	60/438 (13.7)	326/2478^b^ (13.2)	1^NS^, 2–3 *
High BP in pregnancy [n (%)]	53/2283 (2.3)	81/2484 (3.3)	11/438 (2.5)	70/2478 (2.8)	NS
Women who delivered (n)	2107	2297	378	2152	
Stillborn [n (%)]	21/2085 (1.0)	24/2285 (1.1)	3/372 (0.8)	36/2126 (1.7)	NS
Male offspring [n (%)]	1080/2083 (51.8)	1189/2285 (52.0)	192/376 (51.1)	1157/2116 (54.7)	NS
Congenital abnormalities [n (%)]	33/2092 (1.6)	34/2283 (1.5)	10/378 (2.6)	39/2131 (1.8)	NS
Live born, normal singletons (n)	2009	2204	348	2015	
Gestational age at birth [median (range)]	39.6 (28.3–45.4)	39.6 (30.0–45.0)	39.7 (30.9–42.6)	39.5 (28.6–44.2)	NS
Weighed in 72 h (n)	1663	1951	275	1616	
Mean ± SD birthweight (g) (range)	2940 ± 455 (900–4600)	2991 ± 460 (800–4900)	2828 ± 436 (1200–4100)	2891 ± 439 (1200–4270)	1 **, 2 *, 3 **
Proportion LBW [n (%)]	205/1663 (12.3)	203/1951 (10.4)	46/275 (16.7)	222/1616 (13.7)	NS
Median (range) newborn neurological optimality score	16 (6–20)	17 (4–20)	16 (12–20)	17 (8–20)	NS
Neonatal death [n (%)]	29/2064 (1.4)	31/2261 (1.4)	7/369 (1.9)	34/2090 (1.6)	NS

NS: not significant (after Bonferroni adjustment); BP: blood pressure; LBW: low birthweight.

a p-value: ^1^neither vs areca only; ^2^neither vs smokers; ^3^neither vs areca and smoking.

b Maternal death before pregnancy outcome in one women in each group. * p < 0.001; ** p < 0.05.

Of the 6934 pregnancies where the outcome was delivery, there were 2.7% (n = 190) home births with missing data for live birth (n = 66), gender (n = 74) and congenital abnormalities (n = 50). The proportion of male infants, congenital abnormalities and stillbirths was the same for areca users, non-users, smokers and users of both (Table 2).

Gestational age was available for all of the 6576 live born, normal, singletons and birth weight was available for 83.7% (5505) of the same infants, as some were weighed after 72 h of birth. The mean difference in birth weight between areca nut users and non-users was 51 g (95% CI 21–80 g) (p = 0.001). Heavy smokers had babies with a significantly lower mean birthweight than those born to occasional smokers [2851 ± 433 g (range 1200–4200 g) vs 2896 ± 441 g (range 1200–4270 g); p = 0.037], but there was no significant difference in mean birthweight of babies born to heavy areca users compared with occasional users [2960 ± 458 g (range 1300–4380 g) vs 2940 ± 451 g (range 800–4900 g); p = 0.292].

Newborn neurological optimality score was not significantly different between the groups (Table 2) and no increase in hypotonia, tremor or startle response was observed, which might be expected if babies suffered withdrawal symptoms (data not shown).

Areca nut use was not associated with LBW on univariate or multivariate analysis (Table 3) and was also not associated with anaemia after adjustment for covariates (Table 4).

**Table 3:** Risk factors associated with low birthweight (LBW)

Variable		LBW (%)		p-value (univariate)	Adjusted OR (95% CI) (n = 6586)
		Yes	No		
Areca nut user	No	251/1938 (13.0)	1687/1938 (87.0)	0.264	Ref.
	Yes	425/3567 (11.9)	3142/3567 (88.1)		NS
Primigravida	No	414/4222 (9.8)	3808/4222 (90.2)	<0.001	Ref.
	Yes	262/1283 (20.4)	1021/1283 (79.6)		2.26 (1.85–2.76)
First ANC visit after trimester 1	No	397/3509 (11.3)	3112/3509 (88.7)	0.004	Ref.
	Yes	279/1996 (14.0)	1717/1996 (86.0)		1.47 (1.21–1.78)
Malaria infection	No	548/4660 (11.8)	4112/4660 (88.2)	0.002	Ref.
	Yes	128/845 (15.1)	717/845 (84.9)		NS
Smoker	No	408/3614 (11.3)	3206/3614 (88.7)	0.002	Ref.
	Yes	268/1891 (14.2)	1623/1891 (85.8)		1.54 (1.27–1.86)
Female infant	No	322/2894 (11.1)	2572/2894 (88.9)	0.007	Ref.
	Yes	352/2603 (13.5)	2251/2603 (86.5)		1.36 (1.14–1.64)
Mean ± SD gestational age at birth (weeks)		37.8 ± 2.3	39.6 ± 1.2	<0.001	1.97 (1.85–2.09)
Mean ± SD maternal weight at booking^a^ (kg)		45 ± 6	48 ± 7	<0.001	1.08 (1.06–1.10)

Ref.: reference group; NS: not significant; ANC: antenatal clinic.

a Booking refers to the first ANC visit to register the pregnancy.

**Table 4:** Risk factors associated with anaemia at delivery

Variable		Anaemic at delivery (%)		p-value (univariate)	Adjusted OR (95% CI) (n = 6881)
		Yes	No		
Areca nut user	No	425/2459 (17.3)	2034/2459 (82.7)	0.031	Ref.
	Yes	858/4422 (19.4)	3564/4422 (80.6)		NS
Smoker	No	758/4370 (17.3)	3612/4370 (82.7)	<0.001	Ref.
	Yes	525/2511 (20.9)	1986/2511 (79.1)		NS
Malaria infection	No	979/5628 (17.4)	4649/5628 (82.6)	<0.001	Ref.
	Yes	304/1253 (24.3)	949/1253 (75.7)		1.47 (1.21–1.78)
Multigravida	No	220/1563 (14.1)	1343/1563 (85.9)	<0.001	Ref.
	Yes	1063/5318 (20.0)	4255/5318 (80.0)		1.56 (1.33–1.84)
Anaemia at first ANC visit	No	857/5870 (14.6)	5013/5870 (85.4)	<0.001	Ref.
	Yes	426/1011 (42.1)	585/1011 (57.9)		4.13 (3.56–4.78)
First ANC visit after trimester 1	No	694/4178 (16.6)	3484/4178 (83.4)	<0.001	Ref.
	Yes	589/2703 (21.8)	2114/2703 (78.2)		NS

Ref.: reference group; NS: not significant; ANC: antenatal clinic.

## 4 Discussion

Areca nut use alone was not associated with any adverse effects on maternal or neonatal outcomes in pregnant Karen and Burmese women, in contrast to all,^4^, ^9^, ^13^, ^17^, ^18^ except one,^11^ previous publications on the same subject. As far as we are aware, this is the largest cohort of areca use recorded in pregnant women (n = 4963) in the published literature. As reported previously in this population, smoking (cheroots) was a significant risk factor for adverse neonatal outcomes.^21^ Smoking and areca nut use in this population did not result in worse outcomes than smoking alone, lending support to the lack of adverse findings in users of areca only.

Certain adverse associations with areca nut use in pregnancy (LBW^9^, ^10^, ^12^, ^13^, ^17^ and reduced male births^13^) may have resulted from the small sample size and data selection not controlling for parity,^13^ malaria^9^, ^10^, ^11^ or other substance use.^12^, ^13^ However, the lack of significant adverse associations of areca nut in this cohort of pregnant women may be due to differences in the way the nut is prepared and consumed across Asia. The majority of Karen and Burmese pregnant women consume a low number of ripe nuts per day, use *P. betel* Linn*.* leaf and do not add tobacco, whilst Papuans and Taiwanese use a higher number of unripe nuts per day.^5^, ^8^, ^11^, ^12^, ^13^, ^27^

Consistent with two of three prior publications, no relationship with anaemia was apparent when other potential causes were controlled for.^9^, ^10^ Areca nut use has been associated with reduced ovulation and increased risk of miscarriage,^28^ however no increase in miscarriage was observed in this large cohort. This was further supported by finding a dose-dependent increase in miscarriage with smoking but not with areca nut use.

Data on neurological testing of newborns in resource-poor settings is limited.^26^ In contrast to the hypotonia in areca users reported by García-Algar et al.,^4^ there was no increased rate of hypotonia in the cohort presented here. Overall, we were unable to detect an effect of smoking or areca on neurological optimality scores. This is not unexpected as these women continue to consume these products post-partum.

These data were collected over a long period of time (1997–2006), but the 2010 audit suggests the trend of women consuming areca nut during pregnancy continues. It would have been useful to collect data on how each woman prepares and consumes her betel quid. This lack of detail can be generalised to all the publications to date on areca nut use in pregnancy. Further research on areca nut use would benefit from the inclusion of the type of areca nut (ripe or unripe), estimated number of nuts per day, contents of the quid including tobacco and use of the *P. betel* leaf recently reported to have cancer preventive effects,^29^ and whether the woman chews, spits or swallows. Nevertheless, the daily consumption rate that was recorded in the same way for areca and smoking in this cohort did show dose–response relationships for adverse outcomes with heavy smoking but not with heavy areca nut use. This further supports the validity of a lack of adverse effects with areca nut use in this population.

## 5 Conclusion

Although no harmful effects for the mother or neonate following areca nut use during pregnancy were observed, this does not negate the serious negative long-term effects seen in chronic users. A public health initiative to reduce smoking in pregnancy is of greater priority than one to reduce areca nut consumption in this population.

## Authors’ contributions

RM and FN conceived the study; ALC, VIC, MKP, MP, JW, MvV, RM and FN designed the study; ALC, VIC, SJL, FN and RM analysed and interpreted the data; ALC, VIC, MKP, MP, JW and RM drafted the manuscript; ALC, VIC, SJL, MvV, FN and RM critically revised the manuscript for intellectual content. All authors read and approved the final manuscript. RM is guarantor of the paper.

## Funding

Shoklo Malaria Research Unit (Mae Sot, Thailand) is part of the Mahidol–Oxford University Research Unit supported by the Wellcome Trust of Great Britain.

## Competing interests

None declared.

## Ethical approval

Ethical approval for this study was given by Oxford Tropical Research Ethics Committee (reference: OXTREC 28-09).
